# Evaluate the side effect associated with COVID-19 vaccine on adolescents in Riyadh, Saudi Arabia: A cross-section study

**DOI:** 10.15537/smj.2022.43.11.20220493

**Published:** 2022-11

**Authors:** Jehad A. Aldali, Fahad T. Alotaibi, Glowi A. Alasiri, Renad A. Almesned, Aroob M. Alromih, Afnan M. Almohandes, Shahad F. Alsenidi

**Affiliations:** *From the Department of Pathology (Aldali), from the Department of Physiology, College of Medicine (Alotaibi), from the Department of Biochemistry (Alasiri), and from the College of Medicine (Almesned, Alromih, Almohandes, Alsenidi), Imam Mohammad Ibn Saud Islamic University, Riyadh, Kingdom of Saudi Arabia.*

**Keywords:** COVID-19, BNT162 vaccine, Moderna, Oxford-AstraZeneca, Saudi Arabia, side-effects, adolescents

## Abstract

**Objectives:**

To investigate the side effects of Pizer- BioNTech mRNA (BNT162b2) and Spikevax (mRNA- 1273) Coronavirus disease 2019 (COVID-19) vaccines on adolescents in Riyadh, Saudi Arabia.

**Methods:**

A cross-sectional study using an online questionnaire was carried out among COVID-19 vaccine adolescent recipients in Riyadh, Saudi Arabia. After receiving at least one dose of each vaccine, general and demographic data were collected, and vaccine-related side effects were evaluated.

**Results:**

The final sample consisted of 604 participants with a majority age group of 16-17 years old. Approximately 89.1% of the study participants were female. Most participants reported pain at the injection site (85.1% 1st dose, 79.8% 2nd dose), feeling tired, and headache (58.6% 1st dose, 64.2% 2nd dose). Moreover, we found that patients who took the first dose and had a chronic disease had 2.4 times higher odds of having menstrual disorder (females) than non-chronic disease patients (*p*=0.03) and 4.5 times higher odds of exhibiting breathing congestion (*p*=0.01). In addition, patients with chronic disease had 2.4 times higher odds of exhibiting muscle and joint pain and dizziness than non-chronic disease patients (*p*=0.01, *p*=0.02). Males were less likely to have dizziness after the first dose than females (OR=0.26, *p*=0.01).

**Conclusion:**

This study investigates the adverse effects of COVID-19 vaccines among adolescents in Riyadh. As a result, this study creates a database to inform people about the risk of experiencing side effects based on their gender, age, and the vaccine type; more investigation is needed to better understand the link between risk factors and the development of adverse effects.

**A** widespread Coronavirus disease 2019 (COVID-19) epidemic was reported in Wuhan in December 2019.^1^ As the illness spread globally, generating a pandemic, severe acute respiratory consequences of the disease were documented.^2,3^ In Saudi Arabia, health officials adopted strict preventative measures and cautious procedures such as prohibiting international flights, closing mosques, schools, and colleges, and putting the country under complete lockdown.^4^

While the virus spread and infected millions, pharmaceutical companies raced to create safe and effective vaccines.^3^ As a result, Pfizer-BioNTech and Moderna developed mRNA vaccines which are considered a new approach to prompt an immune response.^5^ The BNT162b2 (Pfizer/BioNTech) vaccine is formulated as lipid particles, allowing RNA of SARS-CoV-2 S antigen to be delivered into host cells and expressed.^6^ Simultaneously, the National Institute of Allergy and Infectious Diseases (NIAID) Vaccine Research Center and Moderna in Cambridge, Massachusetts, collaborated on developing the mRNA-1273 vaccine. The SARS-CoV-2 full-length spike glycoprotein trimer, S-2P, has been stabilized and modified to contain two proline substitutions at the top of the central helix in the S2 subunit in this vaccine. The mRNA is encapsulated in lipid n-anoparticles at a 0.5 mg/ml concentration and diluted with normal saline to achieve the final vaccine concentrations.^6,7^ On the other hand, AstraZeneca has developed viral vector vaccines using an adenovirus that contains chimpanzee DNA and has not been exposed to human populations. Therefore, it does not generate an immune response toward the virus, only to the viral protein encoded in host DNA.^5^

As part of its remarkable efforts to restrict the spread of the SARS-CoV-2 virus, Saudi Arabia launched an early immunization program to vaccinate 70% of the population.^8^ Pfizer-BioNTech mRNA (BNT162b2), Spikevax (mRNA-1273), and Oxford-AstraZeneca (ChAdOx1 nCoV-19) have been accepted in the Kingdom of Saudi Arabia (KSA).^4^ The vaccinations were first approved for selected high-risk individuals such as healthcare professionals and older people with chronic conditions. Then the vaccines were made available to all adults.^4^ Lastly, Pfizer-BioNTech and Moderna were licensed for use on adolescents aged 12 to 17.^9^ Our study aimed to investigate the side effect of Pfizer-BioNTech mRNA (BNT162b2) or Spikevax (mRNA-1273) COVID-19 vaccines on adolescents in Saudi Arabia. The study will help identify the side effects on adolescents.

## Methods

A recent cross-sectional study was carried out in Riyadh, Saudi Arabia, between December 2021 and January 2022 using an online survey. On the fourth day after vaccination, the survey was sent to all adolescents who received the Pfizer-BioNTech or Moderna vaccines in the Riyadh Region of Saudi Arabia. A bilingual (Arabic and English) questionnaire was generated online using Google forms and sent to participants via social media (e-mails and WhatsApp groups). Additionally, an e-mail was created and used to improve communication between study researchers and participants. The survey tool was designed in Arabic/English and reviewed by a panel of professionals who provided feedback on various survey items and subsequently revised based on their recommendations. Participants were directed to a page with a fully comprehensive description of the purpose of the study before they were asked to agree to a mandatory electronic consent form containing data on voluntary participation and anonymity.

The Institutional Review Board committee reviewed and approved this research at Imam Mohammad Ibn Saud Islamic University project number 167-2021, dated December 20, 2021.

We excluded all participants who declined to participate, were not vaccinated against COVID-19, or received a vaccine other than Pfizer-BioNTech or Moderna. The data was uploaded and saved into a safe Excel file when the participant completed the survey. Raosoft (Raosoft: 206-525-4025, US) was used to calculate the sample size.

The structured survey was divided into 2 parts: i) The first part was designed to capture general information regarding participants, such as gender, age, chronic conditions, and whether or not they were infected with SARS-CoV-2. ii) The second part was focused on information concerning the COVID-19 vaccination, including the type and date of the vaccine, whether it was the first or second dosage, common adverse effects associated with the vaccine, and the vaccine’s side effects timing and length.

Many symptoms were listed in the survey, including feeling tired and headache, pain at the injection site, muscle and joint pain and feeling unwell, high temperature and chills, dizziness, vomiting, breathing congestion, menstrual irregularities, and itchy skin or rash. Participants were asked on any side effects they experienced on days 1 to 4 after vaccination. In addition, in the first 4 days after receiving the vaccination, participants were asked to describe the severity of each symptom, and the degree of symptoms ranged from mild to severe. Participants were also asked if pain relievers relieved symptoms. Also, participants were asked regarding the average time it took for their symptoms to appear and how long it lasted.

Saudi Arabia’s Ministry of Health plans to vaccinate 100% of the population (around 8 million people). A sample size of 385 people was enough to obtain 95% confidence and a 5 % margin of error. To estimate the relationship between one or more independent variables and a binary outcome variable logistic regression was used in this study. Binomial logistic regression was performed to determine the effects of gender and chronic diseases on the likelihood that patients have side effects after coronavirus vaccine doses.

### Statistical analysis

The statistical analysis was performed using Statistics Package Social Science for Windows, version 28 (IBM Corp., Armonk, N.Y., USA).

## Results

In total, the study included 604 participants (89.1% females and 10.9% males). The majority of participants (58.1%) were in the age group of 16-17 years old, and 258 (41.9%) were between 12 and 15 years. Most participants (92.4%) reported that they were in good health, and 46 (7.6%) reported that they were ill either with a general disease (5.1%) at the time of the survey or chronic disease (2.5%). A total of 110 (18.2%) participants reported a SARS-CoV-2 infection prior to receiving the first dose of the vaccine, and 8 (1.3%) reported previous/current smoking. The summary of the study as shown in Table 1.

**Table 1:** - The general demographic characteristics of the participants and medical history.

Variables	n	%
***Gender***		
Male	66	10.9
Female	538	89.1
***Age groups***		
12-15	253	41.9
16-17	351	58.1
***Nationality***		
Saudi	455	75.3
Non- Saudi	149	24.7
***Occupation***		
Student	596	98.6
Not student	8	1.4
***Health status***		
Good health	558	92.4
Health problem	31	5.1
Chronic disease	15	2.5
Diagnosed with COVID-19 prior to receiving the first dose of the COVID-19 vaccine	110	18.2
Not diagnosed with COVID-19 prior to receiving the first dose of the COVID-19 vaccine	494	81.8
Smoker/former smoker	8	1.3
Non-smoker	596	98.7
Receiving antimicrobial	9	1.5
Not receiving antimicrobial	595	98.5

Table 2 summarizes the reported side effects of the first and second doses of Pfizer/BioNTech and Moderna vaccines. The majority of participants received Pfizer/BioNTech vaccine in both the first (98.2%) and second dose (92.1%). As for Moderna, only 11 (1.8%) received the first dose, and 39 (6.5%) received the second dose.

**Table 2:** - Participants who experienced side effects, as well as the amount of doses administered (N=604).

Side effect	First dose n (%)	Second dose n (%)
***Type of vaccine***		
BNT162b2 (Pfizer)	593 (98.2)	556 (92.1)
Moderna	11 (1.8)	39 (6.5)
Missing data	0	9 (1.5)
***Side effects after the dose***		
Feeling tired and headache	354 (58.6)	388 (64.2)
Pain on the injection site	514 (85.1)	482 (79.8)
Muscle and joint pain and feeling unwell	201 (33.3)	235 (38.9)
High temperature and shivering in the body	167 (27.6)	232 (38.4)
Dizziness	129 (21.4)	164 (27.2)
Vomiting	24 (4.0)	39 (6.5)
Breathing congestion	26 (4.3)	40 (6.6)
Menstrual disorder	123 (20.4)	106 (17.5)
Skin itching or rash	26 (4.3)	24 (4.0)
No sides effect	35 (5.7)	4 (1.0)
Missing data	0	9 (1.5)
***Side effects were most prominent on the***		
First day	281 (46.5)	320 (53)
Second day	292 (48.3)	253 (41.9)
Third day	10 (1.7)	12 (2)
Other	21 (3.5)	19 (3.1)
***The severity of the side effects***		
Mild	247 (40.9)	186 (30.8)
Moderate	303 (50.2)	264 (43.7)
Severe	54 (8.9)	136 (22.5)
Missing data	0	18 (3.0)
***For how long did the side effect last after vaccination:***		
1–2 days	346 (57.3)	348 (57.6)
3 days	209 (34.6)	176 (29.1)
4 or more	49 (8.1)	60 (9.9)
Missing data	0	20 (3.3)
***Medication usage***		
Take any medication to reduce the severity of the side effects	357 (59.1)	381 (63.1)
Not taking any medication to reduce the severity of the side effects	247 (40.9)	211 (34.9)
Missing data	0	12 (2.0)

The most commonly reported side effects for the first dose were pain at the injection site (85.1%), fatigue and headache (58.6%), and muscle and joint pain (33.3%). Regarding the onset of side effects after the first dose of either SARS-CoV-2 vaccine, most participants reported symptoms the second day (46.5%) after receiving the dose, followed by the first day (48.3%). Thirty-five (5.7%) reported no symptoms after the first dose. The severity of the symptoms, varied between mild (40.9%) and moderate (50.2%). Fifty-four (8.9%) reported severe side effects. Most participants’ symptoms lasted for one to 2 days (57.3%). A significant number of participants (59.1%) took medication to reduce the SARS-CoV-2 vaccine side effects. As for the second dose, the most frequently reported side effects were pain at the injection site (79.8%), fatigue and headache (64.2%), and muscle and joint pain (38.9%). High temperature and shivering in the body (38.4%) along with dizziness (27.2%), and vomiting (6.5%) were slightly more reported with the second dose compared to the first dose, as demonstrated in Table 2. Regarding the onset of side effects after the second dose of either SARS-CoV-2 vaccine, most participants (53%) reported symptoms after the first day of receiving the dose, followed by the second day (41.9%). Interestingly, the stated severity of the symptoms was mostly moderate (43.7%), followed by mild (30.8%). Most participants reported that the symptoms only lasted one to 2 days (57.6%). Additionally, most participants (3.1%) took medication to reduce the SARS-CoV-2 vaccine side effects.

**Table 3:** - Logistic regression (first dose).

Side Effect	Variables	B	*P*-value	Odds ratio	95% CI Lower	95% CI Upper
Muscle and joint pain	Gender	-0.45	0.14	0.64	0.36	1.15
	Presence of chronic disease	0.89	0.01*	2.45	1.24	4.83
Dizziness	Gender	-1.35	0.01*	.26	0.10	0.66
	Presence of chronic disease	0.87	0.02*	2.39	1.16	4.94
Breathing congestion	Gender	-1.26	0.22	0.28	0.04	2.16
	Presence of chronic disease	1.50	0.01*	4.46	1.56	12.73
Menstrual disorder	Gender	NA	NA	NA	NA	NA
	Presence of chronic disease	0.87	0.03*	2.38	1.12	5.10

Assessment is based on patients with chronic disease compared to patients without chronic disease. Gender assessment is males compared to females. *Significant, CI: confidence interval, B: coefficients, NA: not applicable

**Table 4:** - Logistic regression (second dose).

Side effect	Variables	B	*P*-value	Odds Ratio	95% CI Lower	95% CI Upper
Feeling tired and headache	Gender	0.58	0.06	1.78	0.98	3.24
	Presence of chronic disease	-0.71	0.04*	0.49	0.25	0.97
Breathing congestion	Gender	0.33	0.49	1.39	0.55	3.49
	Presence of chronic disease	1.33	0.00*	3.77	1.53	9.28

Assessment is based on patients with chronic disease compared to patients without chronic disease. Gender assessment is males compared to females. *Significant, CI: confidence interval, B: coefficients

Binomial logistic regression was performed to determine the effects of gender and chronic diseases on the likelihood that patients have side effects after coronavirus vaccine doses. The logistic regression model was statistically significant (*p*=0.014). Patients with chronic disease had 2.4 times higher odds of exhibiting muscle and joint pain after the first dose than non-chronic disease patients (*p*=0.01).

Other side effects were also investigated. The logistic regression model was statistically significant for dizziness (*p*<0.01). Males were less likely to have dizziness after the first dose than females (OR=0.26, *p*=0.01). Patients with chronic disease had 2.4 times higher odds of exhibiting dizziness after the first dose than non-chronic disease patients (*p*=0.02). The logistic regression model was statistically significant for breathing congestion, (*p*=0.02). Patients with chronic disease had 4.5 times higher odds of exhibiting breathing congestion after the first dose than non-chronic disease patients (*p*=0.01).

For menstrual disorder logistic analysis, the *p*-value was significant (*p*<0.01). Patients who took the first dose and had chronic disease had 2.4 times higher odds of having menstrual disorder than non-chronic disease patients (*p*=0.03).

For patients who took the second dose, the logistic regression was significant only when investigating chronic disease in patients who felt tired, had a headache after the dose (*p*=0.02), and who had breathing congestion (*p*=0.02). Chronic disease patients were less likely to feel tired or have headaches after a second dose than non-chronic disease patients (OR=0.49, *p*=0.04). Patients with chronic disease had 3.7 times higher odds of exhibiting breathing congestion after the second dose than non-chronic disease patients (*p*<0.01).

## Discussion

In December 2019, Wuhan was hit by a widespread Coronavirus disease 2019 (COVID-19) pandemic.^1^ As the disease spread globally, causing a pandemic, significant acute respiratory complications were reported.^3^ Health officials in Saudi Arabia have implemented stringent preventative measures and cautious processes.^4^

While the virus spread and affected millions, pharmaceutical companies raced to develop safe and effective vaccines.^3^ The immunizations were initially approved for high-risk persons such as healthcare workers and elderly people with chronic problems. Following that, the immunizations were made available to all adults. Finally, Pfizer-BioNTech and Moderna have been approved for use in adolescents aged 12 to 17.^9^

Vaccines are widely regarded as the most successful public health intervention because they prevent and control infectious diseases, thereby lowering mortality. However, reactions to vaccination may occur, just as with other medications. The majority of these reactions are neither severe nor common. Individual faith in vaccines vary significantly and is influenced by various factors, including vaccine information, religious or political beliefs, potential related hazards, and social and economic circumstances. Furthermore, it has been demonstrated that individuals estimate vaccine risk differently than professionals.^8^

This study was designed to investigate the short-term side effects associated with Pfizer-BioNTech mRNA (BNT162b2) or Spikevax (mRNA-1273) COVID-19 vaccines for first and second doses on adolescents in Riyadh, Saudi Arabia. Data was collected by an online questionnaire from adolescents who received COVID-19 vaccines in Riyadh, Saudi Arabia. We found that patients who took the first dose and had chronic disease had 2.4 times higher odds of having menstrual disorder (for adolescent females) than non-chronic disease patients (*p*=0.03) and 4.5 times higher odds of exhibiting breathing congestion than non-chronic disease patients (*p*=0.01). In addition, patients with chronic disease had 2.4 times higher odds of exhibiting muscle and joint pain and dizziness than non-chronic disease patients (*p*=0.01, *p*=0.02). Males were less likely to have dizziness after the first dose than females (OR=.26, *p*=0.01). After the second dose, patients with chronic disease were 3.7 times more likely to exhibit breathing congestion than non-chronic disease patients (*p*=0.01).

Moreover, we noticed that chronic disease patients were less likely to feel tired or have headaches after the second dose than non-chronic disease patients (OR=0.49, *p*=0.04). In the current study, pain at the injection site was the most common side effect (85.1% after dose 1, 79.8% after dose 2). Likewise, previous studies showed that the most common side effect related to the BNT162b2 dose was pain at the injection site.^10,11^ We found that feeling tired and having headaches were the second most frequent side effects (58.6% after dose 1, 64.2% after dose 2). Similarly, fatigue and headache were the second most prevalent in the previous study.^10,11^ Unlike other studies, menstrual disorder was reported as a side effect.

### Study limitations

Because of variances in interpretation and tolerance levels from patient to patient, the results of this questionnaire are self-reported by people receiving the vaccine and not clinically validated by physicians. Also, when responding to the survey on the fourth day following immunization, participants were liable for recall bias, which hampered the accurate recall of events. Using a subjective scale rather than an objective criterion to identify the degree of symptoms, such as mild, moderate, or severe, may have resulted in heterogeneity in participant responses. Furthermore, the sample for this study was taken from only one location (Riyadh region). As a result, the findings may not be applicable in a different area. Furthermore, various confounders may impact how the results are interpreted.

In conclusion, evaluating the side effects associated with the COVID-19 vaccine on adolescents showed that the most common side effect among adolescents was pain at the site of injection. Headache and fatigue were also reported as prevalent adverse effects. This study identified menstrual disorder as a side effect, unlike other investigations.

As a result, this study creates a database to inform people about the risk of experiencing side effects based on their gender, age, and the type of vaccine they receive. However, more investigation is needed to understand better the link between risk factors and the development of adverse effects.

## References

[1] Hong KH , Lee SW , Kim TS , Huh HJ , Lee J , Kim SY , et al. Guidelines for laboratory diagnosis of coronavirus disease 2019 (COVID-19) in Korea. Ann Lab Med 2020; 40: 351–360. 32237288 10.3343/alm.2020.40.5.351 PMC7169629

[2] Mesev EV , LeDesma RA , Ploss A. Decoding type I and III interferon signalling during viral infection. Nat Microbiol 2019; 4: 914–924. 30936491 10.1038/s41564-019-0421-x PMC6554024

[3] Cucinotta D , Vanelli M. WHO declares COVID-19 a pandemic. Acta Biomed 2020; 91: 157. 32191675 10.23750/abm.v91i1.9397 PMC7569573

[4] Alhazmi A , Alamer E , Daws D , Hakami M , Darraj M , Abdelwahab S , et al. Evaluation of side effects associated with COVID-19 vaccines in Saudi Arabia. Vaccines (Basel) 2021; 9: 674. 34207394 10.3390/vaccines9060674 PMC8235009

[5] Mascellino MT , Di Timoteo F , De Angelis M , Oliva A. Overview of the main anti-SARS-CoV-2 vaccines: mechanism of action, efficacy and safety. Infect Drug Resist 2021; 14: 3459. 34511939 10.2147/IDR.S315727 PMC8418359

[6] Meo S , Bukhari I , Akram J , Meo A , Klonoff DC. COVID-19 vaccines: comparison of biological, pharmacological characteristics and adverse effects of Pfizer/BioNTech and Moderna Vaccines. Eur Rev Med Pharmacol Sci 2021: 1663–1669. 33629336 10.26355/eurrev_202102_24877

[7] Anderson EJ , Rouphael NG , Widge AT , Jackson LA , Roberts PC , Makhene M , et al. Safety and immunogenicity of SARS-CoV-2 mRNA-1273 vaccine in older adults. N Engl J Med 2020; 383: 2427–2438. 32991794 10.1056/NEJMoa2028436 PMC7556339

[8] Adam M , Gameraddin M , Alelyani M , Alshahrani MY , Gareeballah A , Ahmad I , et al. Evaluation of post-vaccination symptoms of two common COVID-19 vaccines used in Abha, Aseer Region, Kingdom of Saudi Arabia. Patient Prefer Adherence 2021;15: 1963-1970. 10.2147/PPA.S330689 PMC8434920 34522089

[9] Public Health Authority. Interim guidelines for the use of SARS-CoV-2 vaccine. [Updatyed 2021 Aug 31; Accessed date?]. Available from: https://covid19.cdc.gov.sa/professionals-health-workers/interim-guidelines-for-the-use-of-sars-cov-2-vaccine/

[10] Frenck Jr RW , Klein NP , Kitchin N , Gurtman A , Absalon J , Lockhart S , et al. Safety, immunogenicity, and efficacy of the BNT162b2 Covid-19 vaccine in adolescents. N Engl J Med 2021; 385: 239–250. 34043894 10.1056/NEJMoa2107456 PMC8174030

[11] Hause AM , Gee J , Baggs J , Abara WE , Marquez P , Thompson D , et al. COVID-19 Vaccine Safety in Adolescents Aged 12-17 Years - United States, December 14, 2020-July 16, 2021. MMWR Morb Mortal Wkly Rep 2021; 70: 1053–1058. 34351881 10.15585/mmwr.mm7031e1 PMC8367318

